# Maternal Nutrition During Gestation Alters Histochemical Properties, and mRNA and microRNA Expression in Adipose Tissue of Wagyu Fetuses

**DOI:** 10.3389/fendo.2021.797680

**Published:** 2022-02-01

**Authors:** Yi Zhang, Konosuke Otomaru, Kazunaga Oshima, Yuji Goto, Ichiro Oshima, Susumu Muroya, Mitsue Sano, Sanggun Roh, Takafumi Gotoh

**Affiliations:** ^1^ Faculty of Agriculture, Kagoshima University, Kagoshima, Japan; ^2^ Kuju Agricultural Research Center, Kyushu University, Taketa, Japan; ^3^ Joint Faculty of Veterinary Medicine, Kagoshima University, Kagoshima, Japan; ^4^ Western Region Agricultural Research Center, National Agriculture and Food Research Organization (NARO), Oda, Japan; ^5^ Institute of Livestock and Grassland Science, National Agriculture and Food Research Organization (NARO), Tsukuba, Japan; ^6^ Department of Nutrition, School of Human Cultures, The University of Shiga Prefecture, Hikone, Japan; ^7^ Graduate School of Agricultural Science, Tohoku University, Sendai, Japan

**Keywords:** maternal nutrition, Wagyu fetus, adipose tissue, gene expression, histochemical property

## Abstract

We hypothesized that maternal low or high nutrition would give unique effects to morphological and molecular dynamics in adipose tissue of fetus of fatty breed Wagyu (Japanese Black) cattle which produce highly marbled beef. This study aimed to determine the effects of maternal energy intake in Wagyu cows, during gestation on fetal adipose tissue development, histochemical properties, and gene and microRNA (miRNA) expression. Cows were allocated to one of two nutritional energy groups: 120% (HIGH) or 60% nutritional requirements of (LOW). Fetuses (n = 6 per treatment) were removed from pregnant cows by cesarean section at fetal age 260 ± 8 days and euthanized. Subcutaneous adipose tissue (SAT), thoracic cavity visceral adipose tissue (TVAT), and perirenal adipose tissue (PAT) were collected for analysis. In histochemical analysis, in SAT and PAT, HIGH fetuses had greater diameter of adipocytes than LOW fetuses (P<0.05). Only in SAT, LOW fetuses had more Leptin (LEP) mRNA and tended to have more Peroxisome Proliferator-Activated Receptor gamma (PPARG) CCAAT-enhancer-binding proteins alpha (CEBPA) and Glucose transporter (GLUT) 4 mRNA(P<0.10). In all SAT, TVAT, and PAT, LOW fetuses had higher levels of the brown adipose tissue (BAT) biomarkers Uncoupling Protein (UCP) 1 and PPARG coactivator (PGC) 1α mRNA than HIGH fetuses (P<0.08). Meanwhile, in the other adipose tissue, LOW fetuses had lower PPARG, CEBPA, and Zinc Finger Protein (ZFP) 423 (in TVAT and PAT), FASN (in TVAT), LEP and GLUT4 mRNA (in PAT; P<0.10). In particular, in TVAT and PAT, LOW fetuses exhibited lower expression of WAT biomarkers (PPARG and ZFP423). Differential expression of various miRNAs related to adipogenesis between the LOW and HIGH fetuses was detected in an adipose tissue-specific manner (P<0.10). Based on adipose tissue-specific effects of maternal nutrition, these findings suggested that poor maternal nutrition in Wagyu cattle increased BAT development in SAT, TVAT and PAT, while elevated maternal nutrition stimulated fetal SAT development compared with that of TVAT and PAT.

## Introduction

Through its major contribution as a source of protein in people’s diets, beef plays an important role in human health. To improve meat quality-related factors such as juiciness and flavor, intramuscular adipose tissue is crucial (1, 2). However, adipose tissue in carcasses, that is, SAT, renal adipose tissue, and intermuscular adipose tissue, is basically useless or wasted adipose tissue from the perspective of human consumption. Actually, we partly use Wagyu adipose tissue as a material for processed beef in Japan, however a huge amount of wasted adipose tissue is still abandoned. Wagyu (Japanese Black) cattle have not only a greater percentage of intramuscular fat but also a greater mass of carcass adipose tissue than European cattle (3). Although Wagyu is a unique animal model regarding obesity and intramuscular fat accumulation, we would like to shift the focus on its development from increasing intramuscular fat to instead reducing wasted adipose tissue. However, the molecular mechanism behind the accumulation of such adipose tissue in cattle is unknown.

Adipose tissue is scattered throughout the body but comprises 5% to 35% of cattle body mass, depending on age, genotype, and nutrition (4). In terms of the anatomically distinct sites where adipose tissue develops, there are three major sites of accumulation, visceral, subcutaneous, and intermuscular, which are further subdivided into smaller depots defined by anatomical location. The formation of discernible adipocytes begins mid-gestation in beef cattle (4–6). Prior and Laster (7) confirmed that the maternal period during mid- to late gestation in cattle is crucial for adipose tissue development. Additionally, during fetal muscle development, a small portion of the progenitor cells differentiate into adipocytes, which also form intramuscular fat and marbling in the offspring (8). Some studies have demonstrated that the manipulation of maternal nutrition, including over- and undernutrition during gestation, impacts on adipose tissue development and the expression of adipogenesis marker genes in fetuses (4, 9, 10). This suggests that maternal nutrition during gestation plays an important role in adipose tissue development in fetuses.

There are two types of adipose tissue in mammals, white adipose tissue (WAT) and brown adipose tissue (BAT), which have markedly different morphological roles and biological functions (11). In newborns, BAT is essential for ensuring effective adaptation to the extrauterine environment, and the growth of both WAT and BAT during gestation is largely dependent on the supply of nutrients from mother to fetus (12). There are differences of depots place between rodents (interscapular), large mammals and human (around the central organs and supraclavicular region; 12). Change in maternal nutrition at defined stages of gestation would ultimately have long-term adverse effects on the offspring by modifying normal profiles of adipose development. For example, suboptimal maternal nutrition during early to mid-gestation was reported to result in excess macrophage accumulation and the onset of insulin resistance in an adipose tissue depot-specific manner in offspring (12).

At the molecular level, many important factors involved in adipogenesis have been found (PPARG, CEBPA, Stearoyl-CoA desaturase (SCD), Fatty acid synthase (FASN), Fatty acid binding protein (FABP) 4, LEP, TNFα, and ZFP423) (13–17), and in recent years factors involved in the development of brown adipocytes (Uncoupling protein (UCP) 1, PR/SET domain (PRDM) 16, and PGC1α have also been identified (18). Factors related to adipocyte development and metabolism (IGF1, IGF2 GLUT4, INSR, IRS1 PI3K, AKT1, AKT2, and mTOR1) have been considered (19–22). Furthermore, as another factors affecting these genes expression, miRNAs (miRNA-15b, 16 b, 19b, 27b, 33a, 101, 130a, 148a, 152, 196a, 204, 296-3p, and 378) have been noticed (23–33). It has been still unclear how maternal nutrition affects these factors in fetal adipose tissues of Wagyu cattle and its differences among different adipose depots.

We also previously demonstrated that Wagyu cows fed diets with reduced [60% of nutritional requirement: Japan Feeding Standard for Beef Cattle (34;JSFBC)] and slightly greater than required nutritional content (120% of nutritional requirement: JSFBC) from pre-conception to gestational day 260 produced fetuses with phenotypic differences, including fetuses from the latter group having greater adipose tissue mass (2.12-fold), muscle mass (1.42-fold), bone mass (1.24-fold), and fetal body weight (1.39-fold; 35). Several ruminant groups were used to examine the impacts of under- and overnutrition on fetal development; few phenotypic differences in fetuses were identified, although there were differences in gene expression in muscle (36–40). Fetuses with different phenotypes were obtained by different maternal nutrition during gestation (35), these differences might alter the metabolic rate of the whole fetus and change adipose tissue metabolism. We hypothesized that inadequate maternal energy status would alter fetal adipose tissue development in an adipose tissue depot-specific manner in Wagyu fetuses. The objective of this study was thus to determine the consequences of higher or lower maternal energy status throughout gestation on Wagyu fetuses as follows: 1) on the morphology of adipocytes in SAT, and PAT; and 2) on the expression of genes and miRNAs related to growth, adipogenesis, and glucose metabolism in SAT, TVAT, and PAT.

## Materials and Methods

### Animals, Diets, and Experimental Design

The animal study was reviewed and approved by Kagoshima University Animal Care and Use Committee (A18007). Written informed consent was obtained from the owners of the animals for their participation in this study. The experimental details were previously reported elsewhere (35). Briefly, multiparous Wagyu cows (n = 32) were obtained from Kagoshima University Iriki farm (n = 12) and Western Region Agricultural Research Center (n = 20). The cows were randomly assigned to two dietary treatment groups matched for body mass: diets formulated to meet either 60% (LOW) or 120% (HIGH) of their Japan Feeding Standard for Beef Cattle (34)-predicted energy requirements using formula feed. All cows were housed in a drylot, and diets were individually provided twice daily for 2 months prior to and throughout gestation using stanchions that locked each cow in until all feed had been consumed.

The total mixed ration consisted of whole-crop silage, composed of rice plants, dried timothy grass, rye straw, brown rice, beer lees, sugar cane pellets, tofu lees, soy sauce cake, sugar cane bagasse, rice bran, corn steep liquor, condensed sweet potato distillers’ solubles, rice trienol, calcium, and water. The final crude nutrient composition of mixed feed, on a dry-matter basis, was 56.1% NDF, 36.0% ADF, 11.1% ash, 8.00% crude protein, 0.60% calcium, and 0.30% phosphate. The metabolizable energy provided by the feed was 8.56 MJ/kg dry matter.

All cows were synchronized using a controlled internal drug release device (Easybreed, InterAg Co. Ltd., Hamilton, New Zealand). All cows were inseminated with frozen male-sorted semen from the same sire (Yurikatsuyasu, Kedaka line) to produce half-brothers and to minimize other influencing effects except for nutritional status. After breeding, six cows from each group became pregnant.

### Slaughter and Sample Collection

Maternal body weight was measured every month from the start of the study until cows were transported to Kagoshima University Veterinary Teaching Hospital on day 260 ± 8.3 of gestation. Fetuses were obtained by cesarean section and euthanized. The fetal body weight, and weights of carcass muscle, carcass bone, and adipose tissue depots including SAT, TVAT, and PAT were measured as described by Zhang et al. (35).

### Sample Processing

SAT was collected from total adipose tissue between skin and the outermost parts of skeletal muscle from the right carcass to measure the weight. SAT samples for histochemical and molecular analysis were taken from adipose tissue located around between forelimb and body trunk. TVAT was collected from the thoracic and visceral cavity of the right-side carcass, and PAT was taken from adipose tissue covering the left kidney. The fetal adipose tissue was dissected free of other connective tissue and these samples for histological analysis were immediately covered with Tissue Tek (tissue freezing medium; Sakura Fine Technical, Tokyo, Japan), rinsed in ice-cold saline, snap-frozen in liquid nitrogen, and stored at −80°C until use.

### Histochemical and Immunohistochemical Analyses

A 1-cm^3^ core of adipose tissue from SAT, TVAT and PAT, immediately covered with tissue freezing medium (Tissue Tek; Sakaura Fine Technical, Tokyo, Japan), snap-frozen in liquid nitrogen, and stored at −80°C until analysis. Adipose tissue samples were sectioned at 10-µm thickness using a cryostat microtome CM3050 S (Leica, Bensheim, Germany). These sections were fixed by 10% formaldehyde and stained by using Harris Modified Hematoxylin (Fisher Scientific, Fair Lawn, NJ, USA) and Eosin Y (EMD Chemicals, Gibbstown, NJ, USA).

Rabbit polyclonal antibody to human Ucp1 (ab10983) was obtained from Abcam (Cambridge, MA, UK) to examine immunolocalization in the SAT, TVAT, and PAT. According to the manufacturer, this antibody was predicted to recognize bovine Ucp1 (41). Endogenous peroxidase was blocked using BLOXALL^™^ Endogenous Peroxidase and Alkaline Phosphatase Blocking Solution (Vector Laboratories, Inc., Burlingame, CA, USA) for 10 min at room temperature. The sections were washed with PBS and treated using VECTASTAIN ABC Kit, Peroxidase (Rabbit IgG) (Vector Laboratories, Inc.). In accordance with the manufacturer’s instructions, normal goat serum blocking solution was applied for 20 min at room temperature. After washing with PBS, the sections were incubated with the anti-Ucp1 antibody (diluted 1:400) overnight at 4°C. The sections were then washed with PBS and incubated with a biotinylated goat anti-rabbit secondary antibody for 30 min at room temperature. After washing with PBS, the sections were incubated with peroxidase-conjugated streptavidin for 30 min at room temperature. After washing with PBS, the DAB substrate kit (Nichirei Biosciences, Tokyo, Japan) was applied to the sections for 5 min at room temperature, followed by counterstaining with hematoxylin. The sections were then dehydrated and mounted. The experiments were repeated at least three times and the positive staining was reproducibly detected. The sections were captured with an BZ-X800 ALL-IN-ONE fluorescence microscope (Keyence, Tokyo, Japan) under the same microscope objective (30×), and five randomly chosen fields were taken per section for a total of 20 images per animal. Images were randomly selected for analysis, the diameter of adipocytes based on the average of maximum dimension of the long axis and that of the axis perpendicular to the long axis was calculated for at least 50 cells per field area, and at least 250 adipocytes were measured per animal *via* image analysis using CELL image analysis software (Keyence, Tokyo, Japan) and the cross-sectional area (CSA) of adipocyte was calculated by using the average of the diameter.

### Total RNA Extraction and Real-Time qPCR

Total RNA from each adipose tissue was extracted from less than 100 mg of tissue using miniRNeasy Lipid Tissue Kit (Qiagen, Germantown, MD, USA), in accordance with the manufacturer’s instructions. Total RNA samples were quantified using a spectrophotometer (ND-1000; NanoDrop, Wilmington, DE, USA). The purity of RNA (A_260_/A_280_) for all samples was above 1.9 and the RNA was stored at −80°C until cDNA synthesis. Total RNA (500 ng) from each individual calf and tissue was reverse-transcribed with the High-Capacity cDNA Reverse Transcription Kit (Life Technologies Inc., Carlsbad, CA, USA), in accordance with the manufacturer’s instructions. The RT products (cDNA) were stored at −30°C for relative quantification by PCR.

The primers were designed using Primer Express 3.0 with a minimum amplicon size of 80 bp (when possible, amplicons of 100–200 bp were chosen), and aligned against publicly available databases using BLASTN (Basic Local Alignment Sequence Tool for Nucleic Acid) at the website of the National Center for Biotechnology Information (NCBI; Bethesda, MD, USA; **Table 1**). Before qPCR, primers were tested in a 20 µl PCR reaction using the same protocol as described for qPCR except for the final dissociation protocol. For primer testing, we used a universal reference cDNA (RNA mixture from four different bovine tissues) to ensure identification of the desired genes. A total of 5 μl of the PCR product was run in a 2% agarose gel stained with ethidium bromide (2 µl). The remaining 15 µl was cleaned using the QIAquick PCR Purification Kit (Qiagen). Only those primers that did not present as a primer-dimer, had a single band at the expected size in the gel, and had the right amplification product (verified by sequencing) were used for qPCR. The accuracy of each primer pair was also evaluated by the presence of a unique peak during the dissociation step at the end of quantitative PCR (qPCR).

Real-time PCR analysis was performed in triplicate using 100 ng of cDNA in 96-well fast plates using the SYBR Fast Master Mix ABI Prism (D-Mark Biosciences, Toronto, Canada) and the Step-One Plus Real-time PCR system (Life Technologies Inc.). A blank sample and a minus RT were added to control for nonspecific amplification. Relative standard curves, made from a serial dilution of pooled cDNA from the tissue of interest and ranging from 20 to 0.02 ng, were used to determine the relative quantity of each sample. The amplification efficiency for each gene was determined using serial dilution of tissue-specific cDNA and was found to be 100 ± 10% for all genes. The resulting qPCR amplicons were also sequenced to confirm their identity. For each tissue, two to four endogenous controls were tested and the best individual or combination of endogenous control was chosen using NormFinder. Therefore, Ribosomal protein L32 (RPL32) and Ribosomal protein S18 (RPS18) were used as endogenous controls (**Table 1**) to correct for RNA extraction and reverse-transcription efficiency in the adipose tissues (SAT, TVAF, and PAT, respectively). The endogenous controls were also tested for any treatment effect and were found to be stable among samples within each tissue type, confirming their usefulness as suitable endogenous controls. Sequence-specific products were identified by generating a melting curve in which the Cycle Threshold (CT) value represented the cycle number at which a fluorescent signal was statistically greater than the background. The relative mRNA expression was quantified using the 2^–ΔΔCt^ method and thereby the fold change was calculated (**Supplementary Tables**).

**Table 1:** Primer sequences for gene expression measured by real-time PCR.

	Gene		Sequence 5’--3’	GenBank Accesion	Product Size (bp)
Peroxisome proliferator-activated receptor gamma	*PPARG*	Fwd	ACCACCGTTGACTTCTCCAG	NM_181024.2	137
		Rev	ACAGGCTCCACTTTGATTGC		
CCAAT/enhancer-binding protein alpha	*CEBPA*	Fwd	GCTGACCAGTGACAATGACC	NM_176784.2	109
		Rev	CTTGACCAGGGAGCTCTCG		
Stearoyl-CoA desaturase (delta-9-desaturase)	*SCD*	Fwd	CGACCTAAGAGCCGAGAAGC	NM_173959.4	195
		Rev	GCAGCACTATTCACCAGCCAG		
Fatty acid synthase	*FASN*	Fwd	CTACCAAGCCAGGCAGGTC	NM_001012669.1	226
		Rev	GCCATTGTACTTGGGCTTGT		
Fatty acid binding protein 4	*FABP4*	Fwd	ACAGGAAAGTCAAGAGCATCGT	NM_174314	235
		Rev	TGGACAACGTATCCAGCAGA		
Leptin	*LEP*	Fwd	TGACATCTCACACACGCAGTC	XM_010804453.3	114
		Rev	ATCGCCAATGTCTGGTCCAT		
Tumor Necrosis Factor α	*TNFα*	Fwd	AAGCATGATCCGGGATGTGG	NM_173966.3/ XM_027524120.1	180
		Rev	GACTGCTCTTCCCTCTGGGG		
Uncoupling protein 1	*UCP1*	Fwd	AAACAGAAGGGCCAGTGAAA	NM_001166528.1	220
		Rev	TGCAGTCTGACCTTGACCAC		
PR/SET Domain 16	*PRDM16*	Fwd	CCTTCCCGGGTCCTTACCTA	XM_015475185.2	152
		Rev	CAGGTGGGCAGGTGTGATAG		
PPARG coactivator 1 α	*PGC1α*	Fwd	TGCAGTACACATCAGCCTCA	NM_177945.3	95
		Rev	TGCCAGGAGTTTGGTTGTGAT		
Zinc finger protein 423	*ZFP423*	Fwd	CGCTCGGTGAAAGTTGAAGA	NM_001101893.1	216
		Rev	CTGACAGTGATCGCAGGTGT		
Insulin-like growth factor 1	*IGF1*	Fwd	GATGCTCTCCAGTTCGTGTG	NM_001077828	141
		Rev	CTCCAGCCTCCTCAGATCAC		
Insulin-like growth factor 1 receptor	*IGF1R*	Fwd	CAAAGGCAATCTGCTCATCA	NM_001244612	139
		Rev	CAGGAAGGACAAGGAGACCA		
Insulin-like growth factor 2 receptor	*IGF2*	Fwd	CCAGCGATTAGAAGTGAGCC	NM_174087.3	95
		Rev	AGACCTAGTGGGGCGGTC		
Insulin-like growth factor 2 receptor	*IGF2R*	Fwd	GCAATGCTAAGCTTTCGTATTACG	NM_174352	188
		Rev	GGTGTACCACCGGAAGTTGTATG		
Insulin receptor	*INSR*	Fwd	CCTATGCCCTGGTGTCACTT	XM_002688832	114
		Rev	GCTGCCTTAGGTTCTGGTTG		
Insulin receptor substrate 1	*IRS1*	Fwd	TGGACATCACAGCAGAATGAAGA	XM_003585773.5	287
		Rev	CATGTGGCCAGCTAAGTCCT		
Phosphoinositide-3-kinase	*PI3K(R2)*	Fwd	AACCGAGAGATCGACAAGCG	NM_174576.2	99
		Rev	TTCTGAGTGAGCCACACAAGG		
Protein kinase B	*AKT1*	Fwd	TGAAGACTTTCTGCGGGACC	NM_173986.2	141
		Rev	CCTGGTTGTAGAAGGGCAGG		
AKT serine/threonine kinase 2	*AKT2*	Fwd	ACGAGGAAGGAGTAAAGCGA	NM_001206146.1	148
		Rev	AGCCAGCCTTCTTTGATGACA		
Mechanistic target of rapamycin kinase	*mTOR1*	Fwd	CCTTGGCACAACAGTGCATC	XM_002694043.6	285
		Rev	AGGTCCTCATGTCCTCGTGA		
Glucose transporter 4	*GLUT4*	Fwd	GCTTCCAACAGATCGGCTCTG	NM_174604.1	174
		Rev	CCAGCCAGGTCTCATTGTAGC		
Reference genes	*RPS18*	Fwd	TTCCAGCACATCTTGCGAGT	NM_001033614.2	178
		Rev	TCACACGTTCCACCTCATCC		
	*RPL 32*	Fwd	GCCATCTCTGACTCGGCATC	NM_001034783.2	169
		Rev	TTGAATCTTCTGCGCACCCT		

### Quantitative RT-PCR Analysis for MicroRNA

Total RNA from each adipose tissue was extracted from less than 100 mg of adipose tissue using the miRNeasy Kit (Qiagen, Germantown, MD, USA), in accordance with the manufacturer’s instructions. Following that, all total RNA samples were quantified using a spectrophotometer (ND-1000; NanoDrop, Wilmington, DE, USA). The purity of RNA (A_260_/A_280_) for all samples was above 1.9, and total RNA from each individual calf and tissue was reverse-transcribed with Mir-X miRNA First-Strand Synthesis Kit (Takara Bio USA, Inc.), in accordance with the manufacturer’s instructions. Briefly, cDNAs were reverse-transcribed from 100 ng of total RNA using 2× mRQ buffer and mRQ enzyme miRNA assay. This was performed in a thermal cycler, in which the tube was incubated for 1 h at 37°C, followed by termination at 85°C for 5 min to inactivate the enzymes. After that, 90 µl of ddH_2_O was added to bring the total volume to 100 µl. Next, the reverse-transcription product was amplified with TB Green qRT-PCR miRNA assay, in accordance with the manufacturer’s instructions, while fluorescence signal was detected with a Plus-one Real-time PCR System Detector^®^ (Applied Biosystems). U6 snRNA was selected as reference miRNA in this study due to its stable expression among all animals and treatments. In the current study, the expression of miRNAs (miR-15b, 16b, 19b, 27b, 33a, 101, 130a, 148a, 152, 196a, 204, 296-3p, and 378) was analyzed (**Table 2**). The relative quantification of miRNA (or miR) was performed using the 2^–ΔΔCT^ method (**Supplementary Tables**).

**Table 2:** Primer sequences for miRNA expression measured by real-time PCR.

Representative miRNA	Accession	Mature Sequence	Forward Primer 5’--3’	Length
bta-miR-15b	MIMAT0003792	20 - uagcagcacaucaugguuuaca - 41	TAGCAGCACATCATGGTTTACA	22
bta-miR-16b	MIMAT0003525	17 - uagcagcacguaaauauuggc - 37	TAGCAGCACGTAAATATTGGC	21
bta-miR-19b	MIMAT0004337	54 - ugugcaaauccaugcaaaacuga - 76	TGTGCAAATCCATGCAAAACTGA	23
bta-miR-27b	MIMAT0003546	61 - uucacaguggcuaaguucugc - 81	TTCACAGTGGCTAAGTTCTGC	21
bta-miR-33a	MIMAT0009294	6 - gugcauuguaguugcauugca - 26	GTGCATTGTAGTTGCATTGCA	21
bta-miR-101	MIMAT0003520	49 - uacaguacugugauaacugaa - 69	GCGCTACAGTACTGTGATAACTGAA	25
bta-miR-130a	MIMAT0009223	55 - cagugcaauguuaaaagggcau - 76	CAGTGCAATGTTAAAAGGGCAT	22
bta-miR-148a	MIMAT0003522	44 - ucagugcacuacagaacuuugu - 65	TCAGTGCACTACAGAACTTTGT	22
bta-miR-152	MIMAT0009238	53 - ucagugcaugacagaacuuggg - 74	TCAGTGCATGACAGAACTTGGG	22
bta-miR-196a	MIMAT0009255	16 - uagguaguuucauguuguuggg - 37	TAGGTAGTTTCATGTTGTTGGG	22
bta-miR-204	MIMAT0004338	23 - uucccuuugucauccuaugccu - 44	TTCCCTTTGTCATCCTATGCCT	22
bta-miR-296-3p	MIMAT0009273	47 - gaggguugggcggaggcuuucc - 68	GAGGGTTGGGCGGAGGCTTTCC	*22*
bta-miR-378	MIMAT0009305	43 - acuggacuuggagucagaaggc - 64	ACTGGACTTGGAGTCAGAAGGC	22
U6-F		ctcgcttcggcagcaca	AACGCTTCACGAATTTGCGT	20

*bta, Bos taurus; miR, microRNA.

### Statistical Analysis

Data were analyzed as a randomized complete block design with cow as the experimental unit. The fixed effect was maternal treatment and the random effect included maternal farm of origin. Data were analyzed using SAS (Version 9.2, SAS Institute, Inc. Carey, NC, USA) and treatment means were compared using the PDIFF option. Expression differences of mRNA and microRNA between three kinds of adipose tissue depots (SAT, TVAT, and PAT) were determined using Dunnett’s modified Tukey-Kramer pairwise multiple comparison test (DTK). Differences were considered significant at *p* < 0.05 and trends were considered at 0.05 < *p* < 0.10.

## Results

### Phenotypic Data

The fetuses obtained in this experiment at slaughter (day 260 ± 8.3) showed marked differences between the LOW and HIGH groups, as reported by Zhang et al. (35; **Table 3**). The body, carcass muscle, carcass adipose tissue, and carcass bone weights of LOW fetuses were lower than those of HIGH fetuses (*p* < 0.05). Regarding the specific adipose depots, the weights of SAT (3.03-fold), TVAT (1.47-fold), and PAT (1.45-fold) were greater in HIGH fetuses than in LOW fetuses, as shown in **Table 3** (35).

**Table 3:** Effects of LOW or HIGH maternal nutrition of Wagyu cows during the entirety of gestation on the fetal BW and fat weight.

Item	Weight (g)		*p*-value	HIGH/LOW^5^	Ratio ^3^(%)		*p*-value
	LOW^1^	HIGH^2^			LOW	HIGH	
Animals, No.	6	6			6	6	
Fetal BW, g	23390.0	32653.0	0.0018	1.396			
fat weight^4^, g	333.0	708.0	0.0004	2.126	5.4	8.1	0.023
TVAT^6^	25.5	37.6	0.018	1.475	0.5	0.5	0.937
SAT	41.5	125.8	0.006	3.032	0.8	1.6	0.032
PAT^7^	82.2	119.2	0.006	1.450	0.4	0.4	0.769
other fat	183.8	425.4					

Data are mean ± standard error. ^1,2^Wagyu cows were fed diets providing a low (60%; LOW) or high nutrition level (120%: HIGH), according to the JFSBC (34) nutritional requirements. ^3^Ratio of mass relative to the calculated fetal half-carcass (right side) mass. ^4^Mass calculated for the right half of the carcass with right-side perirenal adipose tissue. ^5^Fold difference between the high-nutrition group and the low-nutrition group for variables with p<0.10. ^6^The weights of adipose tissue of the thoracic or peritoneal cavity on the right side of the carcass. ^7^The adipose tissue surrounding the right and left kidneys. *These data are from Zhang et al. (35).

### Adipose Tissue Morphology

**Figure 1:**
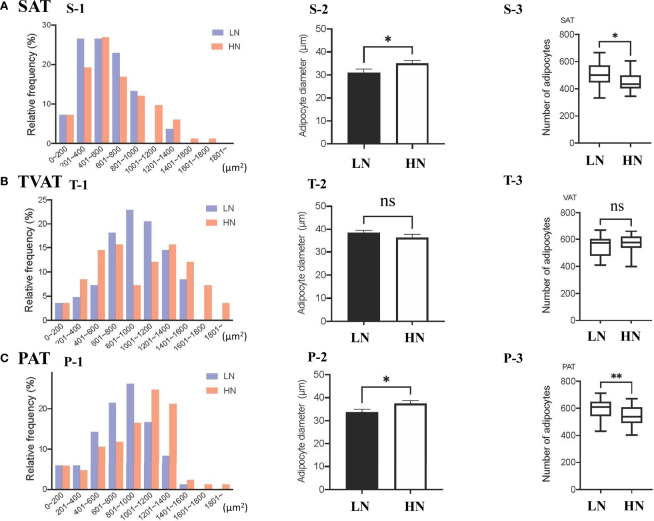
**(A)** Relative frequency distribution of SAT CSA (S-1), diameter (S-2), and number of adipocytes within the same area (S-3) in subcutaneous adipose tissue (SAT) between LOW and HIGH fetuses. **(B)** Relative frequency distribution of TVAT CSA (T-1), diameter (T-2), and number of adipocytes in the same area (T-3) in thoracic cavity visceral adipose tissue (TVAT) between LOW and HIGH fetuses. **(C)** Relative frequency distribution of PAT CSA (P-1), diameter (P-2), and number of adipocytes within the same area (P-3) in perirenal adipose tissue (PAT) between LOW and HIGH fetuses. Fetuses: 260 ± 8.3 days of fetal age. LOW: n=6. HIGH: n=6. Values are means with standard errors. Significant differences between fetal groups are denoted by *p < 0.05 and **p < 0.01.

**Figure 2:**
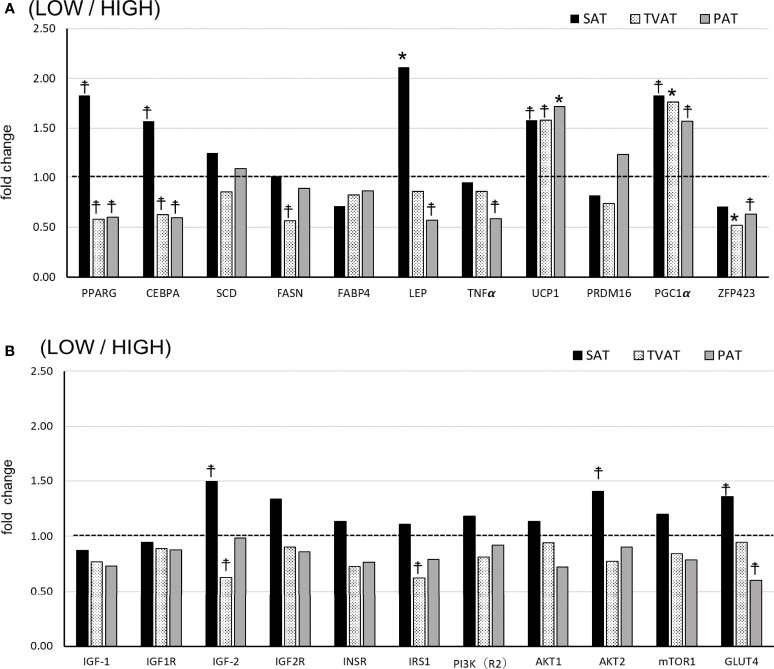
Comparison of fold change of mRNA abundance in subcutaneous (SAT), thoracic cavity visceral (TVAT), and perirenal adipose tissues (PAT) between LOW and HIGH fetuses in Wagyu cattle. **(A)** Adipogenesis including brown adipose tissues (LOW/HIGH), **(B)** growth factors, and glucose metabolism (LOW/HIGH). The horizontal dotted line (at the value “1”) showed the relative mRNA expression of the HIGH fetuses. The foldchange was calculated as the relative expression based on the mean of the target gene Ct value of LOW and HIGH fetuses. Fetuses: 260 ± 8.3 days of fetal age. LOW: n=6. HIGH: n=6. *Significant difference and ^☨^trend (*p* < 0.05 and *p* < 0.1, respectively) between LOW and HIGH fetuses.

**Figure 3:**
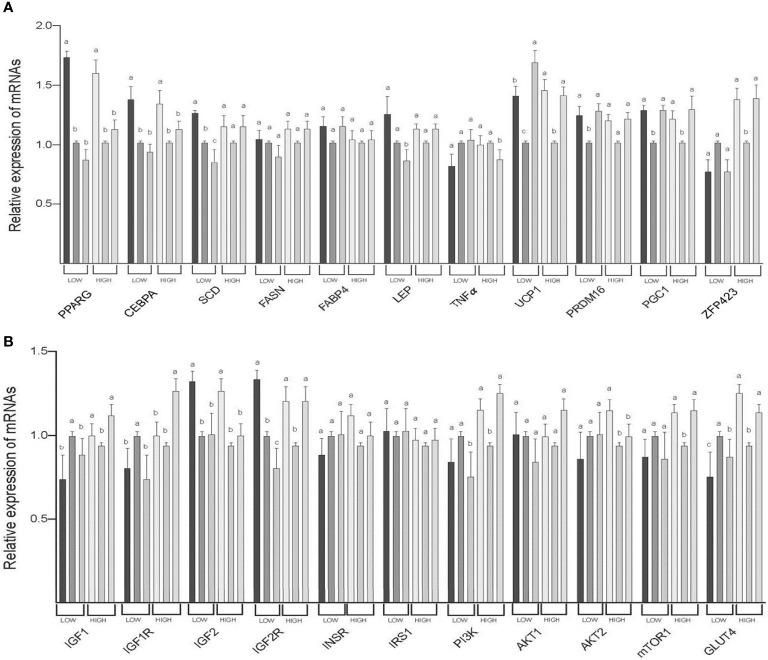
Comparison of mRNA abundance among subcutaneous (SAT), thoracic cavity visceral (TVAT), and perirenal adipose tissues (PAT) in the LOW and the HIGH fetuses in Wagyu cattle. **(A)** Adipogenesis including brown adipose tissues (LOW/HIGH), **(A)** growth factors, and glucose metabolism (LOW/HIGH). Fetuses: 260 ± 8.3 days of fetal age. LOW: n=6. HIGH: n=6. ^a,b,c^Significant difference (*p* < 0.05) among SAT, TVAT, and PAT in the LOW and the HIGH fetuses.

The frequency of adipocytes CSA in SAT showed similar patterns between LOW and HIGH fetuses, however, the relative frequency of 201-400 μm^2^ adipocyte CSA was somewhat higher in LOW fetuses (**Figure 1**, S-1). The frequency of adipocyte CSA in TVAT showed higher frequency of 801-1000 μm^2^ adipocyte CSA in LOW fetuses and, on the other hand, there were two peaks of relative frequencies (601-800 μm^2^ and 1201-1400 μm^2^) in HIGH fetuses (**Figure 1**, T-1). The frequency of adipocyte CSA in PAT showed the highest at 801-1000 μm^2^ in LOW fetuses, and meanwhile at 1001-1200 μm^2^in HIGH fetuses (**Figure 1**, P-1). In SAT (*p* < 0.01) and PAT (*p* < 0.05), the average adipocyte diameter was greater in HIGH fetuses than in LOW ones (**Figure 1**, S-2, P-2). Consistent with these results, the adipocyte number/unit area of SAT (*p* < 0.05) and PAT (*p* < 0.01) was greater in LOW fetuses than in HIGH ones (**Figure 1**, S-3, P-3). There were no differences in adipocyte diameter and adipocyte number/unit area in TVAT between the treatment groups (**Figure 1**, T-1, 2 and 3).

### mRNA Expression

In SAT, LOW fetuses had a higher level of LEP (*p* = 0.045) and tended to have higher PPARG (*p* = 0.078), CEBPA (*p* = 0.098), UCP1 (*p* = 0.072), PGC1 (*p* = 0.053), IGF2 (*p* = 0.098), AKT2 (*p* = 0.087), and GlUT4 (*p* = 0.092) mRNA levels than HIGH fetuses (**Figures 2A, B**).

In TVAT, LOW fetuses had higher PGC1α (*p* = 0.045) and lower ZFP423 (*p* =0.042) mRNA levels, and tended to have a higher UCP1 (*p* = 0.067) mRNA level than HIGH fetuses (**Figure 2A**). Meanwhile, LOW fetuses had lower PPARG(*p* = 0.059), CEBPA (*p* = 0.053), FASN (*p* = 0.086), IGF2 (*p* = 0.069), and IRS1 (*p* = 0.071) mRNA levels than HIGH fetuses (**Figure 2A, B**).

In PAT, LOW fetuses had a higher UCP1 (*p* = 0.046) mRNA level and tended to have a higher PGC1α (*p* = 0.073) mRNA level than HIGH fetuses. Conversely, LOW fetuses had lower PPARG (*p* = 0.066), CEBPA (*p* = 0.052), LEP (*p* = 0.068), TNFα (*p* = 0.079), ZFP423 (*p* = 0.078), and GLUT4 (*p* = 0.054) mRNA levels than HIGH fetuses (**Figure 2A, B**).

The expression of PPARG (both LOW and HIGH), CEBPA (both LOW and HIGH), IGF2 (both LOW and HIGH), and AKT2 (HIGH) mRNAs was greater in SAT than in TVAT and PAT (*p* < 0.05) with no significant difference in the TVAT and PAT (**Figure 3**). The expression of SCD (LOW) and IGF2R (LOW) mRNAs was greatest in SAT and least in PAT (*p* < 0.05). The expression of LEP(LOW), TNFα (HIGH), and PI3K (LOW) mRNAs was greater in SAT and TVAT relative to PAT (*p* < 0.05) without significant difference in the SAT and the TVAT. The expression of UCP1 (LOW) mRNA was greatest in PAT and least in TVAT (*p* < 0.05). The expression of UCP1 (HIGH), PRDM16 (LOW), PGC1 (both LOW and HIGH), ZFP423 (HIGH), IGF1 (HIGH), IGF2R (HIGH), PI3K (HIGH), mTOR1 (HIGH), and GLUT4 (HIGH) mRNAs was greater in SAT and PAT than in TVAT (*p* < 0.05), whereas in the SAT and the PAT, they showed no significant difference. Conversely, the expression of IGF1 (LOW) and IGF1R (LOW) m RNAs was greater in TVAT than in SAT and PAT (*p* < 0.05) without significant difference in the SAT and the PAT. The expression of IGF1R (HIGH) mRNA was greater in PAT than in SAT and TVAT (*p* < 0.05) without significant difference in the SAT and the TVAT. No significant difference was observed between SAT, TVAT and PAT in the expression of other genes in the nutritional treatment groups not shown so far (**Figure 3**).

### Immunohistochemistry

**Figure 4:**
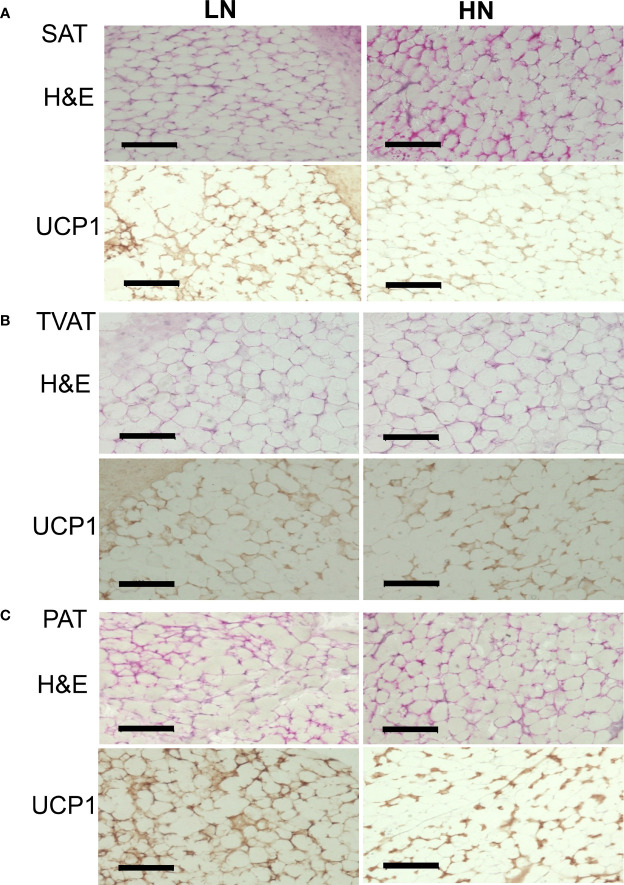
Representative images of H&E staining and immunohistochemical staining of UCP1 in LOW and HIGH fetuses. UCP1: uncoupling protein 1. SAT: subcutaneous adipose tissue (**A**, SAT). TVAT: thoracic cavity visceral adipose tissue (**B**, TVAT). PAT: perirenal adipose tissue (**C**, PAT) in Wagyu fetuses. Fetuses: 260 ± 8.3 days of fetal age. LOW: n=6. HIGH: n=6. Black scale bar represents 100 μm. Magnification: 30×.

Immunohistochemical analysis of SAT, TVAT, and PAT revealed UCP1-positive staining as a marker of brown adipocytes in both LOW and HIGH fetuses (**Figure 4**).

### miRNA Expression

**Figure 5:**
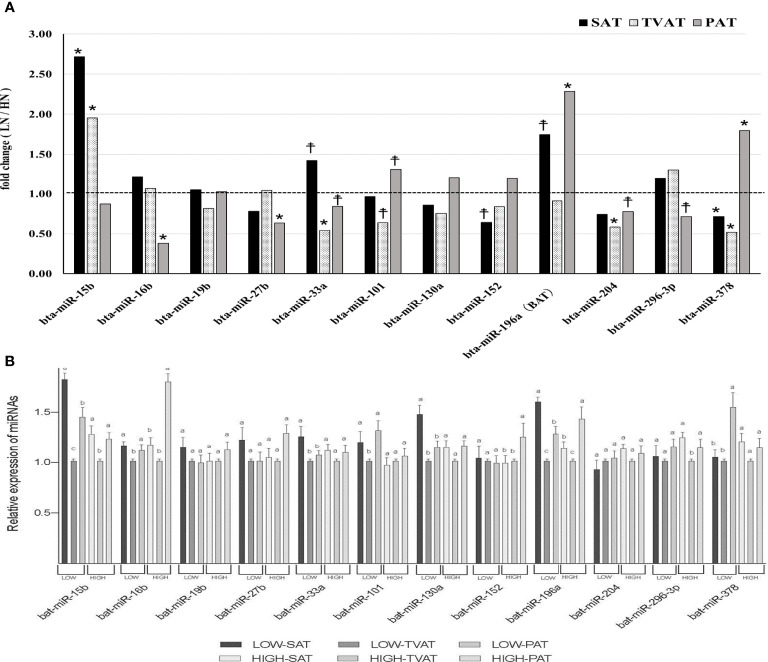
Comparison of miRNAs in adipose tissues. **(A)** Comparison of fold change (**LOW/HIGH**) of miRNA abundance in subcutaneous (SAT), thoracic cavity visceral (TVAT), and perirenal adipose tissues (PAT) between LOW and HIGH fetuses in Wagyu cattle. The horizontal dotted line (at the value “1”) showed the relative miRNA expression of the HIGH fetuses. The foldchange was calculated as the relative expression based on the mean of the target gene Ct value of LOW and HIGH fetuses. **(B)** Comparison of mRNA abundance between SAT, TVAT, and PAT in the LOW and the HIGH fetuses in Wagyu cattle. Fetus age: Fetuses: 260 ± 8.3 days of fetal age. LOW: n=6. HIGH: n=6. *Significant difference and ^☨^trend (*p* < 0.05 and *p* < 0.1, respectively) between LOW and HIGH fetuses. ^a,b,c^ Significant difference (*p* < 0.05) between SAT, TVAT, and PAT in the LOW and the HIGH fetuses.

In SAT, Low fetuses had a higher level of miR-15b (*p* = 0.021) and tended to have higher levels of miR-33a (*p* = 0.057) and miR-196a (*p* = 0.054; **Figure 5**). Conversely, LOW fetuses had a lower level of miR-378 (*p* = 0.042) and tended to have a lower level of miR-152 (*p* = 0.088).

In TVAT, LOW fetuses had a higher level of miR-15b (*p* = 0.011). Meanwhile, LOW fetuses had lower levels of miR-33a (*p* = 0.014), miR-204 (*p* = 0.022), and miR-378 (*p* = 0.009) and tended to have a lower level of miR-101 (*p* = 0.059; **Figure 5A**).

In PAT, LOW fetuses had higher levels of miR-196a (*p* = 0.018) and miR-378 (*p* = 0.047) than HIGH fetuses. Similarly, LOW fetuses tended to have a higher level of miR-101 (*p* = 0.072). Conversely, LOW fetuses had lower levels of miR-16b (*p* = 0.013) and miR-27b (*p* = 0.034) than HIGH fetuses. Simultaneously, LOW fetuses tended to have lower levels of miR-33a (*p* = 0.064), miR-204 (*p* = 0.053), and miR-296-3p (*p* = 0.069; **Figure 5A**).

The expression of miR-33a (LOW) and 130a (LOW) was greater in SAT than in TVAT and PAT (*p* < 0.05) with no significant difference in the TVAT and the PAT (**Figure 5B**). The expression of miR-15b (LOW) and 196a (LOW) was greatest in SAT and least in TVAT (*p* < 0.05). The expression of miR-15b (HIGH), 16b (LOW), 101 (LOW), and 296-3p (HIGH) was greater in SAT and PAT relative to TVAT (*p* < 0.05) without significant difference in the SAT and the PAT. The expression of miR-16b (HIGH), 152 (HIGH), and 378 (LOW) was greater in PAT than in SAT and TVAT (*p* < 0.05) with no significant difference in the SAT and the TVAT. The expression of miR-196a (HIGH) was greatest in TVAT and least in SAT (*p* < 0.05). No significant difference was observed between SAT, TVAT and PAT in the expression of other miRNAs in the nutritional treatment groups not shown so far (**Figure 5B**).

## Discussion

The function of adipose tissue changes with development. In the newborn, BAT is needed to ensure an effective response to the extrauterine environment. Overall adipose tissue mass increases during late gestation, with a mixture of white and brown adipocytes. After that, during postnatal life, some, but not all, adipose depots are replaced by white adipocytes. The changes in maternal nutrition at the mid and the late gestation modify adipose tissue development profiles (12, 42). The gene expression change occurs in an adipose tissue depot-specific manner in offspring born to mothers fed lower nutrition from early to mid-gestation in cattle (43). Maternal low nutrition decreased the expression of Adipocyte Protein (AP) 2 and GLUT4 mRNAs and increased the expression of Cluster of Differentiation (CD) 36 mRNAs in PAT, but not in SAT of offspring in crossbred Angus cattle (43). However, the detailed differential-responses and its relationships of mass and molecular dynamics between SAT, TVAT and PAT in fetuses of different maternal nutrition have not been examined well in cattle. In this study, we demonstrated that lower or higher maternal nutrition during gestation could alter adipose tissue mass, adipocyte size, and gene expression in an adipose-tissue-specific manner in fetuses of Wagyu cattle. In all adipose tissue of LOW fetuses, gene expression analysis indicated enhanced BAT development. In addition, gene expression related to WAT differed between SAT and the other adipose tissues (TVAT and PAT), suggesting the different susceptibility of adipose tissue to maternal nutrition.

There is a need to develop an efficient feeding system because the cost of feeding cattle is increasing. Although the body adipose tissue of beef cattle is not only for energy storage but also an endocrine organ affecting metabolism (44) as well as meat quality and quantity (3), in the beef industry carcass adipose tissue is practically and economically wasted, apart from intramuscular adipose tissue (45). Regulating the development of adipose tissue in cattle could lead to the development of an efficient feeding system. In cows, not only growth but also reproduction and maintenance of body functions require adequate nutrition. Animals use energy and nutrients obtained from feed in various ways. Ferrell and Jenkins (46) reported that, in mature beef cows, maintenance requirements represent approximately 70% to 75% of the total annual energy requirements. In this study, we established two nutritional groups allocated 60% and 120% of nutritional requirements during gestation and identified clear phenotypic differences in fetuses between them (35). Chronic restriction of maternal nutrition has been indicated to reduce basal metabolic rate (47, 48) and to impact fetal development (42, 49). In this study, differences in the development of adipocytes of SAT, TVAT, and PAT between LOW and HIGH fetuses were shown, that namely, adipose tissue mass increased in HIGH fetuses. The expression of UCP1 and PGC1α mRNA increased or tended to increase in SAT, TVAT, and PAT of LOW fetuses and only in SAT of LOW fetuses had more LEP mRNA and tended to have more PPARG, CEBPA, and GLUT4 mRNA. Thus Maternal nutrition would thus strongly affect the accumulation of adipocytes and the activation of their gene expression in the fetus.

As an endocrine organ, adipose tissue is not only involved in energy storage but also acts as a complex, important, and metabolically active part of the body (44). Adipose tissue is scattered throughout the whole body, but makes up 5% to 35% of cattle body mass, which is dependent on age, genotype, and nutrition (4). In adipose tissue, there are three major depot locations, visceral, subcutaneous, and intermuscular depots, which can be further subdivided into smaller depots defined by anatomical location (perirenal and omental) (4). In Wagyu cattle, with growth and fattening, subcutaneous and visceral adipose tissue exhibits particular development that differs from the findings in European breeds such as Holstein, German Angus, and Belgian Blue cattle (3). In mammals including cattle, adipose tissue has been classified into two distinct types: one is white adipose tissue, which has a major function in energy storage; and the other is brown adipose tissue, which is specialized for energy expenditure (4). In recent time, beige adipose tissue, which is a mixture of brown and white adipocyte, was identified. Beige adipocyte is transdifferentiated from WAT and has many similar morphological and functional properties with brown adipocyte (50).

In cattle, the perirenal adipose tissue starts to appear in fetuses from around day 80 of gestation, followed by visceral, subcutaneous, and intermuscular adipose tissue from day 180 of gestation onward, whereas intramuscular adipocytes in fetus muscles starts to develop from mid- and late-gestation (6, 8), and discernible intramuscular adipocytes filled with lipid develop after birth in earnest (4). Considering the results obtained in this study, given the specific functions and different distributions of individual adipose tissue depots, nutritional interventions during gestation could impact on fetal adipose tissue development in diverse ways. However, few studies focusing on ruminants have evaluated the effects of suboptimal maternal nutrition on the development of fetal adipose tissue. Jennings et al. (51) found that maternal high (146% nutrition requirement) and low (72% nutrition requirement) nutrition during mid-gestation did not affect the expression of genes related to adipogenesis in subcutaneous adipose tissue of Angus crossbred fetuses at d 180. These results suggest that fetal growth characteristics are not affected by the level of maternal nutritional manipulation imposed in the study during mid-gestation. While, Long et al. (43) fattened the offspring exposed to 55% maternal nutrition during early gestation, indicated no differences in distribution of subcutaneous and perirenal adipose tissue compared with the control offspring, but the expression of AP2, CD36, and GLUT4 in the perirenal adipose tissue was suppressed in low nutrition group. Regardless of the animal species, the effects of maternal nutrition on fetal development are supposed to be affected by the timing of treatment during gestation (49, 52), although here we manipulated maternal nutrition during the entirety of gestation.

In this study, the adipocyte diameters of SAT and PAT were greater in HIGH fetuses, while there were no differences in adipocyte diameter in TVAT between LOW and HIGH fetuses. This suggested that specific adipose tissue depots showed different responses to suboptimal maternal nutrition during pregnancy. Moreover, it was also suggested that the adipocytes of SAT and PAT were more sensitive to nutritional deficiencies. In calves born from dams fed a restricted level of protein, the mass weight of perirenal adipose tissue, adipocyte size, and lipogenic activities were reported to be similar to those in control calves at birth (53).

**Figure 6:**
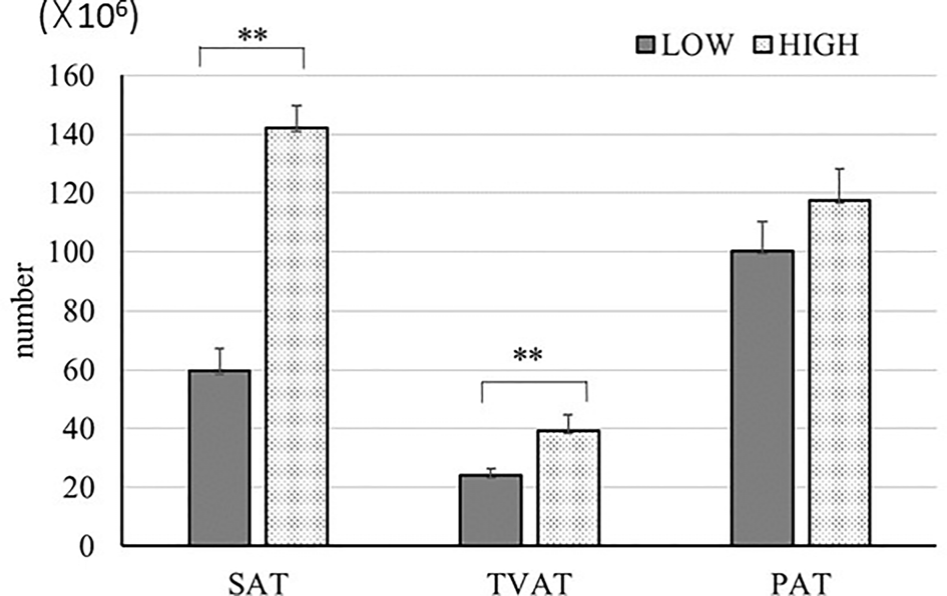
Estimation of adipocyte number in SAT, TVAT, and PAT of LOW and HIGH fetuses. Values were calculated based on the values from adipocyte diameter analysis (**Figure 1**) and data of each adipose tissue mass. Adipose tissue density: 0.92 g/cm^3^ (54). Fetuses: 260 ± 8.3 days of fetal age. LOW: n=6. HIGH: n=6. Values are means with standard errors. Significant differences between fetal groups are denoted by **p < 0.01.

We estimated the number of adipocytes (**Figure 6**) based on the adipocyte diameter and mass of adipose tissue in SAT, TVAT, and PAT (35). In this simulation, we used 0.92 g/cm^3^ as the adipose tissue density (54) and assumed that the adipocytes were spherical for this calculation. The results showed that the numbers of adipocytes in SAT (2.3-fold), TVAT (1.6-fold) were greater in HIGH fetuses than in LOW ones (*p* < 0.01; **Figure 6**). This suggests that maternal nutrition would alter the number of adipocytes in adipose tissue, with SAT and TVAT being particularly sensitive to this. However, the number of adipocytes could be increased after birth by proliferation (55).

In the immunohistochemical observation on UCP1-positive status, SAT, TVAT, and PAT seemed to show greater numbers of brown adipocytes in LOW fetuses than in HIGH fetuses. These findings are supported by the higher UCP1 mRNA expression in SAT, TVAT, and PAT (**Figure 4**). This suggested that maternal overnutrition could suppress brown adipocyte development during gestation. Similar to this result, when the dams consumed a suboptimal amount of nutrients during the final month of gestation, birth weight and adipose tissue mass were reduced, but this is highly likely to reflect a reduction in adipose tissue stores rather than a reduction in the amount of brown adipose tissue in mammals (12). Intriguingly, the reduced amount of adipose tissue present in newborns has a greater capacity to maintain UCP1 as a BAT biomarker, which could be indicative of a protective mechanism against subsequent exposure to some environment with temperature change or an obesogenic environment in human (12). Furthermore, enhanced maternal nutrition from mid- to late gestation could accelerate the activity of glucocorticoids and a series of inflammatory responses in newborns when key inflammatory genes were upregulated (56). However, maternal overfeeding (150% of control) during mid- to late gestation did not affect the weight of perirenal BAT or the adipocyte diameter of fetuses, but increased some adipogenic factor, PPARG, and in lipoprotein lipase, adiponectin, and LEP mRNA expression in PAT (57).

In the adipogenesis, PPARG, CEBPA, SCD, FASN, FABP 4, ZFP423, and LEP have the crucial role (13–17). The changes in the expression of various mRNAs in this study suggested that the adipogenesis of Wagyu fetuses could be susceptible to change due to maternal nutritional status during gestation in a manner dependent on each particular adipose tissue depot. The formation and composition of adipose tissue in cattle are derived from a complex process. They are controlled by multiple parameters including genetic factors, nutritional status, feeding system, species, and sex in cattle (58). In this study, the pattern of gene expression in SAT differed from those in TVAT and PAT. Regarding SAT, HIGH fetuses had 3-fold greater mass than LOW fetuses, while for TVAT and PAT, HIGH fetuses had only 1.4-fold greater mass (35). In addition, SAT from LOW fetuses had greater expression of PPARG, CEBPA, and LEP mRNA, which might suggest that they were trying to develop WAT because the formation of WAT was delayed due to maternal lower nutrition compared with that from HIGH fetuses. Moreover, only in SAT, LOW fetuses tended to have higher levels of IGF2, AK2, and GLUT4 mRNA. Thus, in SAT, depending on the maternal nutritional level, LOW fetuses showed different patterns of mRNA expression related to genes involved in growth and glucose metabolism compared with the findings for TVAT and PAT. Enhanced LEP mRNA expression indicates elevated insulin sensitivity (59), while enhanced GLUT4 expression indicates elevated glucose intake (60). In addition, increased expression of PPARG and CEBPA mRNA in SAT indicates that adipocyte differentiation is activated (61, 62).

Conversely, in TVAT and PAT, LOW fetuses tended to have lower expression of PPARG and CEBPA mRNA and, in PAT, also tended to have lower expression of LEP mRNA, showing the opposite tendency to SAT. SAT is a layer of subcutaneous fat is located between the dermis and the underlying fascia on the outer muscle. SAT is not only serving as a reserve source of energy for the body, but also SAT helps to physically insulate the body from cold (63) and radiates heat in the case of containing BAT in a part of SAT. Moreover, in all of SAT, TVAT, and PAT, LOW fetuses had higher levels of UCP1 and PGC1α mRNA, indicating that brown adipocyte formation was activated in LOW fetuses. Heat-producing adipocytes, brown adipocytes, drive heat production through the close coordination of substrate supply with the mitochondrial oxidative machinery and effectors that control the rate of substrate oxidation (64, 65). Brown adipocyte as a heat production effector specifically expresses UCP1, which is the best characterized marker of BAT (66). With regard to adipose tissue mass, LOW fetuses had only half the carcass mass of HIGH fetuses (35), resulting in the heat-producing ability also being lower. Landis et al. (67) reported the expression of UCP mRNA in the tail-head subcutaneous adipose tissue of cross-bred fetuses though the gene expression level was low. These dynamics of gene expression suggested that, in LOW fetuses, brown adipocytes are activated more for thermogenesis to increase the potential for survival.

In this study, in PAT, LOW fetuses tended to have a lower level of GLUT4 mRNA. Undernutrition during late pregnancy in sheep was reported to reduce the expression of GLUT4 protein in renal adipose tissue and simultaneously generate glucose resistance in offspring at 1 year of age (68). However, the renal and omental adipose tissue mass levels of nutrient restricted group during late gestation were increased compared with those of control group (68). Further research is needed to investigate whether LOW fetuses exhibit an increase in mass later in life. In PAT, lower maternal nutrition increased the levels of PPARG and UCP1 mRNAs in fetuses compared with higher maternal nutrition and a positive correlation between the levels of PPARG and UCP1 mRNAs was reported in sheep (69). These findings were not consistent with our results showing decreased abundance of PPARG mRNA and increased abundance of UCP1 mRNA in LOW fetuses.

Wagyu (Japanese Black) cattle is a unique breed with a high capacity to produce marbled meat. In Wagyu, not only did intramuscular adipose tissue increase 12-fold (2.18% to 26.77%) but also SAT increased 14-fold (3.11% to 44.26%) during fattening (8 to 26 months of age), which contrasts with the finding for PAT (5.25-fold: 4.58% to 24.07%; 3). Conceivably, this ability could be revealed during the fetal development period, and this potential is likely to be influenced by maternal nutrition during gestation. In this study, the sensitivity of fetal SAT to maternal nutrition was quite high.

Notably, studies in mice have shown that ZFP423 is a transcription factor responsible for the adipogenic commitment of progenitor cells (70). The expression of ZFP423 commits progenitor cells to the adipogenic lineage and ensures their differentiation into pre-adipocytes, subsequently inducing PPARG expression, which results in their terminal differentiation (15). The importance of ZFP423 in bovine adipogenesis was further confirmed (16). Shao et al. (71) reported that Fetal development of subcutaneous white adipose tissue is dependent on ZFP423. In this study, in TVAT and PAT, LOW fetuses had a lower expression level of ZFP423 mRNA than HIGH fetuses (*p*<0.10). On the other hand, it was suggested that LOW fetuses activated white adipose tissue formation in SAT because of increased ZFP423 expression.

The brown adipocytes originate primarily from cells in the dermomyotome expressing engrailed 1 (En1), myogenic factor 5 (Myf5), and paired-box protein 7 (Pax7), which can also give rise to muscle cells during the fetal period (72, 73). Thus, the fate of myogenesis during fetal development might be associated with brown adipocytes, which could in turn be affected by maternal nutrition. Although in mouse, Yang et al. (74) previously observed that maternal overnutrition increased the white adipogenesis of progenitors in highly nourished fetuses, while high maternal nutrition during lactation also impaired the thermogenic function of BAT in offspring (75). Therefore, accompanied by lower levels of UCP1, PRDM16, and PGC1α mRNAs in HIGH fetuses, it is suggested that the manipulation of maternal nutrition, especially long-term maternal overnutrition, might adversely affect brown adipose tissue mass and its function in adipose depots of the fetus. 

MicroRNAs have been shown to be important for various biological processes including adipose tissue development. This time we have chosen miRNAs which powerfully affect gene expression related adipogenesis. It has been demonstrated that microRNAs play a critical role in regulating differentiation and function in both WAT and BAT (76, 77). In this study, similar to the results of mRNA expression, differential expression of miRNAs was observed among adipocytes of SAT, TVAT, and PAT (**Figure 5A**). These findings suggested that the manipulation of maternal nutrition throughout gestation could change the miRNA expression dynamics and differentially regulate the development of each adipocyte depot.

miR-15b expression increased in SAT and TVAT of LOW fetuses, but not in PAT. miR-15b has been found to regulate lipid metabolism negatively in adipocytes (regulating DLK1 as a target gene; 25). Increased miR-15b expression in SAT and TVAT of LOW fetuses might reduce lipid metabolism and lead to decreases in lipid content in adipocytes and adipocyte differentiation by reducing the amount of DLK1 (25).

A number of key transcription factors including PPARG and CEBPA/B are known to regulate adipocyte terminal differentiation and lipid metabolism (78), while miRNAs have been demonstrated to regulate adipocyte differentiation through both direct and indirect targeting of these critical transcription factors, as well as their downstream targets (77). PPARG is considered the master regulator of adipocyte differentiation and is a direct target of miR-27a/b (79) and miR-130 (80). Although PPARG mRNA exhibited different expression in adipose tissue among SAT, TVAT, and PAT, miR-27b decreased the expression only in PAT and miRNA-130a did not show any differences in expression in each adipose tissue depot. This suggested that maternal nutrition could affect adipose tissue development, but not through these miRNAs in fetuses. Meanwhile, the inhibition of miR-27b in glucocorticoid-treated mice was found to increase energy expenditure, reduce body weight, and improve the regulation of glucose homeostasis (31). These effects are likely mediated through the targeting of PRDM16, although numerous other factors are involved in promoting BAT function, including PPARA and PPARG coactivator 1-beta (PGC-1β), which have also been identified as targets of miR-27 (81).

Interestingly, miR-196a plays an essential role in BAT progenitor cells and induces the “browning” of WAT, with enhanced expression of BAT genes, inducing PRDM16, UCP1, and PGC-1α (18: rodent). Moreover, miR-196a promotes “browning” by directly binding and suppressing Homeobox C8 (HoxC8), a determinant of white adipogenesis (82: vertebrate; 28: mouse). Because the development of BAT in TVAT of LOW fetuses is promoted with no change of miR-196a expression between LOW and HIGH fetuses, it was suggested that miR-196a was not responsible for promoting BAT development.

miR-378 has been characterized as another positive regulator of BAT (26). In transgenic mice, miR-378 is associated with increased BAT mass and decreased WAT mass, which might be caused by BAT expansion and increased energy expenditure (26). miR-378 was shown to be increased in PAT of LOW fetuses, while UCP1 and PGC-1α mRNA expression was also increased in PAT. It appears that malnutrition could promote BAT development in PAT of LOW fetuses. However, the expression of miR-378 in SAT and TVAT indicated the opposite trend to that in PAT, so it seems that the biological function of miR-378 in PAT differs from those in SAT and TVAT, which is altered by maternal nutrition.

miR-204 showed a lower level in TVAT and PAT of LOW fetuses, but not in SAT. Indeed, miR- has been found to target runt-related transcription factor 2 (RUNX2), which is considered the master regulator of osteoblast differentiation and is one of the main sites of miRNA-mediated adipocyte/osteoblast cell fate determination, resulting in impaired osteogenesis and improved adipogenesis (24). It is suggested that malnutrition during gestation might influence adipose development through downregulating miR-204.

miR-16b regulates metabolism, cell cycle, and inflammatory stress response (83, 84). miR-33a suppresses UCP1 mRNA expression by regulating target genes in BAT (32). In SAT, LOW fetuses retained higher UCP1 mRNA expression although miR-33a expression was higher than that in HIGH fetuses. Meanwhile, there was reasonable interaction between TVAT and PAT, indicating decreased miR-33a and enhanced UCP1 mRNA expression. miR-101 inhibits cell differentiation and induces apoptosis (33). There were different expression patterns of miR-101 expression between TVAT and PAT. miR-152 was previously reported to downregulate preadipocyte proliferation and upregulate differentiation in an *in vitro* experiment using 3T3-L1 (29). Only in SAT, LOW fetuses had a lower abundance of miR-152. This suggests that adipocyte differentiation might be activated more in SAT. miR296-3p enhances angiogenesis and cellular proliferation and conversely downregulates apoptosis (27). Only in PAT, decreased expression of miR-296-3p was observed in LOW fetuses, suggesting that angiogenesis and cellular proliferation might be activated in PAT. However, more recently, updated information regarding these miRNAs has been reported (85). We have to carefully examine the related information and verify the relationship between the obtained data and adipose tissue development in bovine fetuses.

Finally, when comparing the differences in gene expression between adipose tissue depots, 10 genes showed a similar relationship between LOW and HIGH fetuses (**Figure 3**). However, other genes showed different patterns of gene expression between LOW and HIGH fetuses. In particular, the expression of PPARG, CEBPA, and IGF2 mRNA was higher in SAT than in TVAT and PAT in both LOW and HIGH fetuses, and it was speculated that WAT formation and development were enhanced in SAT. On the other hand, UCP1 as a marker of Brown adipose tissue has higher expression in PAT than TVAT, suggesting that PAT has more Brown adipocytes. In general, it was suggested that the molecule dynamics in SAT and PAT were more activated compared with the TVAT. When comparing the differences in miRNA expression between the adipose tissue depots, no differences of half miRNAs expression were observed between SAT, TVAT, and PAT, and the related pattern was different between the LOW and HIGH fetuses (**Figure 5B**). There seems to be many cases of higher miRNA expression in the SAT and PAT than in TVAT. However, further studies are needed regarding these reasons.

## Conclusion

**Figure 7:**
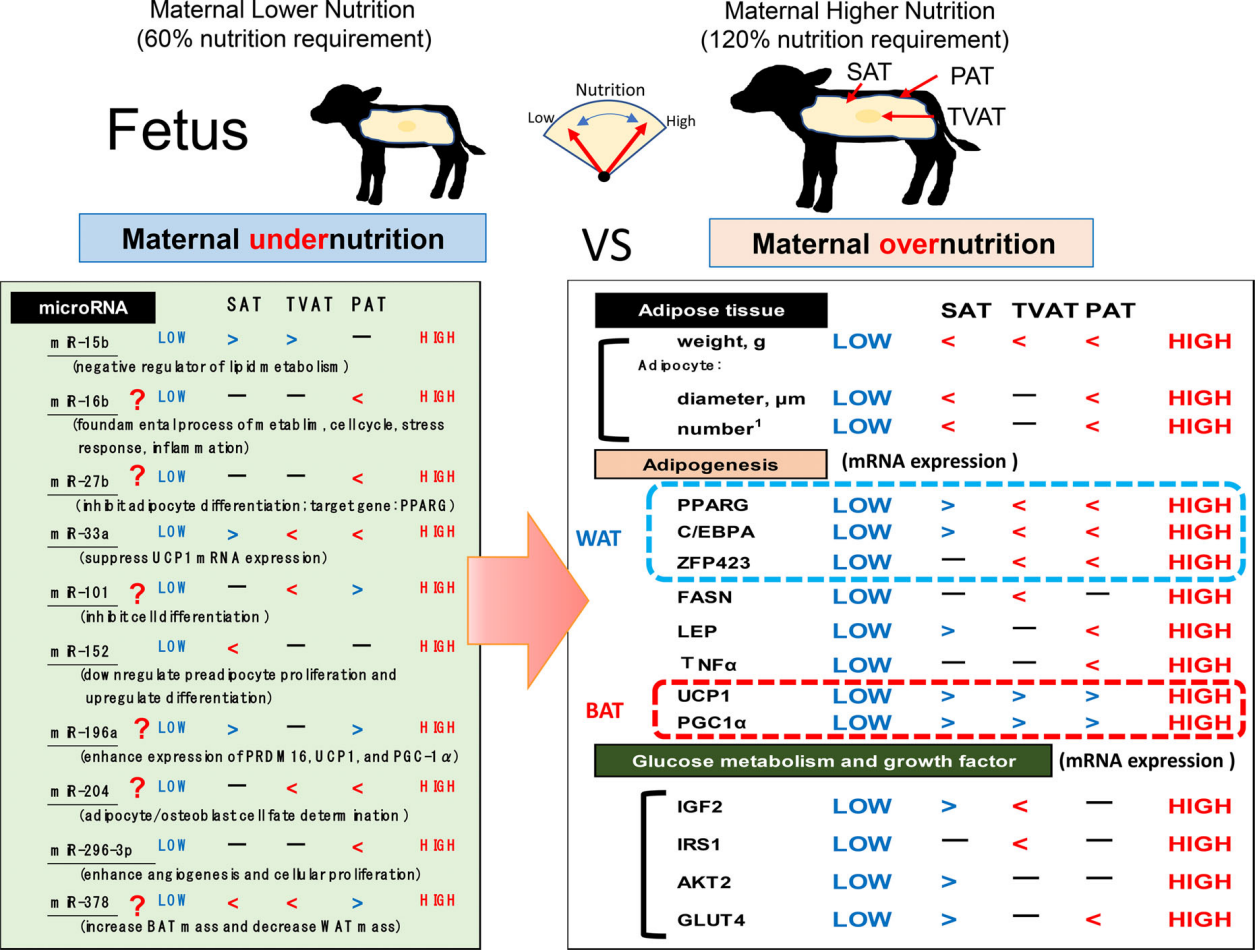
Hypothetical scheme of the influences of maternal under- or overnutrition on the associations between fetal SAT, TVAT, and PAT development in weight, histochemical properties, gene expression (related to adipogenesis, growth factors, and glucose metabolism), and miR expression. Inequality sign is based on *p *< 0.10.

The present study revealed that maternal nutrition during gestation affected the development of adipose tissue by changing not only mass but also mRNA and miRNA expression in an adipose depot-specific manner in fetuses of fatty breed, Wagyu cattle (**Figure 7**). These findings suggest that low maternal nutrition would lead to the development of BAT in fetal adipose tissues and this BAT activation in LOW fetuses might be a priority and delayed WAT formation due to lower nutrition. Intriguingly, more significant differences in mRNA expression were observed in SAT and PAT than in TVAT. Moreover, in SAT and PAT, HIGH fetuses had a greater diameter of adipocytes than LOW fetuses. Meanwhile, the findings also suggested that lower maternal nutrition during gestation could suppress the WAT development in TVAT and PAT, but would accelerate that in SAT. The sensitivity of fetuses to low maternal nutrition in SAT would differ from that in TVAT and PAT. Various miRNAs showed significant differences between the LOW and HIGH fetuses in an adipose tissue-specific manner.

## Data Availability Statement

The data sets presented in this study can be found in online repositories. The names of the repository/repositories and accession number(s) can be found in the article/**Supplementary Material**.

## Ethics Statement

The animal study was reviewed and approved by Kagoshima University Animal Care and Use Committee (A18007).

## Author Contributions

YZ: Conceptualization, methodology, software, writing—original draft preparation. KoO: Surgery management, animal care, data curation/investigation/validation. KaO: Reproduction management, animal care, data curation investigation validation. YG: Reproduction management, animal care, data curation/investigation/validation. IO: Reproduction management, data curation/investigation/validation. SM: Data curation/investigation/validation. MS: Data curation/investigation/validation. SR: Physiological data curation, methodology writing—review and editing. TG: Supervision, conceptualization, funding acquisition, writing—review and editing. All authors contributed to the article and approved the submitted version.

## Funding

This study received funding from Leave a Nest Co., Ltd., the Canon Foundation (R15-0089), Japan Racing Association and Kakenhi (No. 26310312 and19KT0013) from the Japan Society for the Promotion of Science. The authors declare that the funders had no role in study design, data collection and analysis, decision to publish, or preparation of the manuscript.

## Conflict of Interest

The authors declare that the research was conducted in the absence of any commercial or financial relationships that could be construed as a potential conflict of interest.

## Publisher’s Note

All claims expressed in this article are solely those of the authors and do not necessarily represent those of their affiliated organizations, or those of the publisher, the editors and the reviewers. Any product that may be evaluated in this article, or claim that may be made by its manufacturer, is not guaranteed or endorsed by the publisher.
