# Supplementary material for: Maternal Nutrition During Gestation Alters Histochemical Properties, and mRNA and microRNA Expression in Adipose Tissue of Wagyu Fetuses
Source: Front Endocrinol (Lausanne). 2022 Feb 1;12:797680. doi: 10.3389/fendo.2021.797680 (PMC8844027; doi:10.3389/fendo.2021.797680)
Supplement: Supplementary file 1 [file DataSheet_1.pdf]

Supplementary table 1. The data of Ct values regarding mRNA expression in SAT, TVAT, and PAT.

**SAT**	**LOW (n=6)**		**HIGH (n=6)**		**ΔCt (LOW)** = ΔCt(Target - PRS18)	**ΔCt (HIGH)** = ΔCt (Target − PRS18)	**ΔΔCt (LOW − HIGH)** = ΔCt (LOW) − ΔCt(HIGH)	$2^{-\Delta\Delta Ct(LOW-HIGH)}$ **foldchange** (relative expression)	***p* value**
	**Ct (Target) mean**	**Ct (PRS18) mean**	**Ct (Target) mean**	**Ct (PRS18) mean**					
PPARG	24.99	20.11	25.74	19.99	4.88	5.75	-0.87	1.83	0.078
CEBPA	27.45	19.76	28.46	20.12	7.69	8.34	-0.65	1.57	0.098
SCD	24.95	19.99	24.95	19.67	4.96	5.28	-0.32	1.25	0.231
FASN	26.78	20.78	25.77	19.75	6.00	6.02	-0.02	1.01	0.432
FABP4	25.94	20.01	25.23	19.79	5.93	5.44	0.49	0.71	0.273
LEP	23.99	20.18	25.01	20.12	3.81	4.89	-1.08	2.11	0.045
TNFα	24.23	19.19	24.39	19.42	5.04	4.97	0.07	0.95	0.191
UCP1	26.02	19.78	27.02	20.12	6.24	6.90	-0.66	1.58	0.072
PRDM16	26.05	19.89	26.89	21.02	6.16	5.87	0.29	0.82	0.216
PGC1α	23.71	20.11	24.54	20.07	3.60	4.47	-0.87	1.83	0.053
Zfp423	25.22	19.39	25.10	19.77	5.83	5.33	0.50	0.71	0.176
IGF-1	23.82	19.23	24.06	19.67	4.59	4.39	0.20	0.87	0.548
IGF1R	24.38	19.49	24.56	19.75	4.89	4.81	0.08	0.95	0.292
IGF-2	26.99	20.11	27.48	20.02	6.88	7.46	-0.58	1.49	0.098
IGF2R	27.45	19.47	27.84	19.44	7.98	8.40	-0.42	1.34	0.102
INSR	28.58	19.79	28.72	19.75	8.79	8.97	-0.18	1.13	0.231
IRS1	29.89	20.01	29.57	19.54	9.88	10.03	-0.15	1.11	0.179
IGFBP1	27.51	20.31	27.79	20.66	7.20	7.13	0.07	0.95	0.260
PI3K （R2）	25.48	19.56	25.91	19.75	5.92	6.16	-0.24	1.18	0.320
AKT1	26.45	19.99	26.51	19.87	6.46	6.64	-0.18	1.13	0.129
AKT2	25.99	20.01	26.24	19.77	5.98	6.47	-0.49	1.40	0.087
mTOR1	26.31	20.09	26.81	20.33	6.22	6.48	-0.26	1.20	0.177
GLUT4	24.12	20.01	24.56	20.01	4.11	4.55	-0.44	1.36	0.092

**TVAT**	**LOW (n=6)**		**HIGH (n=6)**		**ΔCt (LOW)** = ΔCt(Target - PRS18)	**ΔCt (HIGH)** = ΔCt (Target − PRS18)	**ΔΔCt (LOW − HIGH)** = ΔCt (LOW) − ΔCt(HIGH)	$2^{-\Delta\Delta Ct(LOW-HIGH)}$ **foldchange** (relative expression)	***p* value**
	**Ct (Target) mean**	**Ct (PRS18) mean**	**Ct (Target) mean**	**Ct (PRS18) mean**					
PPARG	24.56	20.89	23.23	20.34	3.67	2.89	0.78	0.58	0.059
CEBPA	26.88	19.73	25.69	19.21	7.15	6.48	0.67	0.63	0.053
SCD	27.48	19.88	26.82	19.45	7.60	7.37	0.23	0.85	0.111
FASN	26.17	19.89	25.12	19.66	6.28	5.46	0.82	0.57	0.086
FABP4	24.07	19.78	23.56	19.55	4.29	4.01	0.28	0.82	0.311
LEP	26.01	21.23	25.77	21.21	4.78	4.56	0.22	0.86	0.397
TNFα	27.41	20.11	27.89	20.81	7.30	7.08	0.22	0.86	0.414
UCP1	28.78	20.33	29.12	20.01	8.45	9.11	-0.66	1.58	0.067
PRDM16	27.12	20.32	26.45	20.09	6.80	6.36	0.44	0.74	0.233
PGC1α	24.17	21.04	25.26	21.31	3.13	3.95	-0.82	1.77	0.045
Zfp423	28.34	20.11	27.41	20.13	8.23	7.28	0.95	0.52	0.042
IGF-1	24.58	20.03	24.38	20.21	4.55	4.17	0.38	0.77	0.127
IGF1R	25.43	19.79	25.12	19.65	5.64	5.47	0.17	0.89	0.258
IGF-2	27.99	20.31	27.22	20.21	7.68	7.01	0.67	0.63	0.069
IGF2R	26.41	20.67	26.33	20.74	5.74	5.59	0.15	0.90	0.151
INSR	25.32	20.08	25.13	20.35	5.24	4.78	0.46	0.73	0.175
IRS1	24.38	20.32	24.27	20.89	4.06	3.38	0.68	0.62	0.071
IGFBP1	24.98	20.33	25.18	20.81	4.65	4.37	0.28	0.82	0.232
PI3K （R2）	27.02	19.38	26.45	19.11	7.64	7.34	0.30	0.81	0.380
AKT1	29.01	20.31	29.39	20.78	8.70	8.61	0.09	0.94	0.490
AKT2	28.91	20.21	29.10	20.77	8.70	8.33	0.37	0.77	0.274
mTOR1	26.99	19.19	27.22	19.67	7.80	7.55	0.25	0.84	0.197
GLUT4	28.01	19.89	28.59	20.55	8.12	8.04	0.08	0.95	0.115

**PAT**	**LOW (n=6)**		**HIGH (n=6)**		**ΔCt (LOW)** = ΔCt(Target - PRS18)	**ΔCt (HIGH)** = ΔCt (Target − PRS18)	**ΔΔCt (LOW − HIGH)** = ΔCt (LOW) − ΔCt(HIGH)	$2^{-\Delta\Delta Ct(LOW-HIGH)}$ **foldchange** (relative expression)	***p* value**
	**Ct (Target) mean**	**Ct (PRS18) mean**	**Ct (Target) mean**	**Ct (PRS18) mean**					
PPARG	27.10	20.31	26.05	19.99	6.79	6.06	0.73	0.60	0.066
CEBPA	25.17	20.56	24.66	20.80	4.61	3.86	0.75	0.59	0.052
SCD	29.79	20.56	29.44	20.09	9.23	9.35	-0.12	1.09	0.224
FASN	26.66	20.23	26.39	20.13	6.43	6.26	0.17	0.89	0.119
FABP4	25.12	20.56	25.23	20.88	4.56	4.35	0.21	0.86	0.291
LEP	26.67	21.21	25.43	20.78	5.46	4.65	0.81	0.57	0.068
TNFα	26.01	20.56	25.56	20.88	5.45	4.68	0.77	0.59	0.079
UCP1	22.09	20.13	23.41	20.67	1.96	2.74	-0.78	1.72	0.046
PRDM16	25.01	20.19	25.78	20.66	4.82	5.12	-0.30	1.23	0.133
PGC1α	28.09	20.41	29.32	20.99	7.68	8.33	-0.65	1.57	0.073
Zfp423	25.22	19.89	24.76	20.09	5.33	4.67	0.66	0.63	0.078
IGF-1	27.12	19.67	27.01	20.01	7.45	7.00	0.45	0.73	0.193
IGF1R	29.91	20.03	30.00	20.31	9.88	9.69	0.19	0.88	0.262
IGF-2	26.82	20.11	27.00	20.31	6.71	6.69	0.02	0.99	0.215
IGF2R	27.84	20.44	27.99	20.81	7.40	7.18	0.22	0.86	0.267
INSR	27.73	19.66	27.56	19.88	8.07	7.68	0.39	0.76	0.231
IRS1	29.57	20.01	29.43	20.21	9.56	9.22	0.34	0.79	0.112
IGFBP1	27.41	19.89	27.13	20.09	7.52	7.04	0.48	0.72	0.147
PI3K （R2）	25.09	19.91	25.11	20.05	5.18	5.06	0.12	0.92	0.427
AKT1	26.71	20.78	26.45	20.99	5.93	5.46	0.47	0.72	0.173
AKT2	26.24	20.11	25.86	19.88	6.13	5.98	0.15	0.90	0.112
mTOR1	26.81	20.01	26.64	20.19	6.80	6.45	0.35	0.78	0.154
GLUT4	24.99	20.89	23.82	20.45	4.10	3.37	0.73	0.60	0.054

Supplementary table 2. The data of Ct values regarding miRNA expression in SAT, TVAT, and PAT

**SAT**	**LOW (n=6)**		**HIGH (n=6)**		**ΔCt (LOW)** = ΔCt(Target - PRS18)	**ΔCt (HIGH)** = ΔCt (Target − PRS18)	**ΔΔCt (LOW－HIGH)** = ΔCt (LOW) − ΔCt(HIGH)	$2^{-\Delta\Delta Ct(LOW-HIGH)}$ **foldchange** (relative expression)	***p* value**
	Ct(Target) mean	Ct(PRS18) mean	Ct(Target) mean	Ct(PRS18) mean					
bta-miR-*15b*	19.31	14.61	19.67	13.53	4.70	6.14	-1.44	2.72	0.021
bta-miR-*16b*	14.97	14.61	14.61	13.97	0.37	0.64	-0.28	1.21	0.193
bta-miR-*19b*	19.93	13.87	19.78	13.65	6.06	6.13	-0.07	1.05	0.337
bta-miR-27b	17.00	14.61	16.00	13.97	2.39	2.04	0.35	0.78	0.216
bta-miR-*33a*	24.50	13.87	24.79	13.65	10.63	11.14	-0.51	1.42	0.057
bta-miR-*101*	18.52	14.61	18.51	14.65	3.91	3.85	0.05	0.96	0.277
bta-miR-*130a*	19.51	14.27	19.49	14.46	5.25	5.03	0.22	0.86	0.772
bta-miR-*152*	22.18	14.27	21.73	14.46	7.91	7.27	0.64	0.64	0.088
bta-miR-*196a*	22.44	14.31	23.33	14.40	8.13	8.93	-0.80	1.75	0.054
bta-miR-*204*	20.88	14.61	20.48	14.65	6.26	5.83	0.43	0.74	0.311
bta-miR-*296-3p*	21.44	14.31	21.79	14.40	7.13	7.39	-0.26	1.19	0.492
bta-miR-*378*	16.85	14.61	16.41	14.65	2.24	1.76	0.48	0.72	0.042

**TVAT**	**LOW (n=6)**		**HIGH (n=6)**		**ΔCt (LOW)** = ΔCt(Target - PRS18)	**ΔCt (HIGH)** = ΔCt (Target − PRS18)	**ΔΔCt (LOW－HIGH)** = ΔCt (LOW) − ΔCt(HIGH)	$2^{-\Delta\Delta Ct(LOW-HIGH)}$ **foldchange** (relative expression)	***p* value**
	Ct(Target) mean	Ct(PRS18) mean	Ct(Target) mean	Ct(PRS18) mean					
bta-miR-*15b*	16.99	14.57	17.71	14.32	2.42	3.39	-0.97	1.95	0.011
bta-miR-*16b*	17.32	14.98	16.17	13.74	2.34	2.43	-0.09	1.07	0.508
bta-miR-*19b*	20.86	14.69	19.62	13.74	6.18	5.88	0.29	0.82	0.267
bta-miR-27b	15.67	14.78	15.27	14.32	0.89	0.95	-0.06	1.04	0.661
bta-miR-*33a*	21.71	14.80	19.77	13.74	6.91	6.03	0.88	0.54	0.014
bta-miR-*101*	20.79	15.02	19.13	14.02	5.76	5.11	0.65	0.64	0.059
bta-miR-*130a*	21.13	15.01	20.03	14.32	6.12	5.71	0.41	0.75	0.191
bta-miR-*152*	21.76	14.63	21.20	14.32	7.13	6.88	0.26	0.84	0.372
bta-miR-*196a*	24.74	14.63	24.30	14.32	10.11	9.98	0.13	0.91	0.235
bta-miR-*204*	20.92	14.63	19.84	14.32	6.29	5.52	0.77	0.59	0.022
bta-miR-*296-3p*	23.42	14.22	24.00	14.43	9.19	9.57	-0.37	1.30	0.424
bta-miR-*378*	15.78	14.63	14.51	14.32	1.15	0.19	0.95	0.52	0.009

**PAT**	**LOW (n=6)**		**HIGH (n=6)**		**ΔCt (LOW)** = ΔCt(Target - PRS18)	**ΔCt (HIGH)** = ΔCt (Target − PRS18)	**ΔΔCt (LOW－HIGH)** = ΔCt (LOW) − ΔCt(HIGH)	$2^{-\Delta\Delta Ct(LOW-HIGH)}$ **foldchange** (relative expression)	***p* value**
	Ct(Target) mean	Ct(PRS18) mean	Ct(Target) mean	Ct(PRS18) mean					
bta-miR-*15b*	18.51	13.19	18.58	13.46	5.31	5.12	0.19	0.87	0.417
bta-miR-*16b*	16.12	13.19	15.87	14.34	2.93	1.53	1.40	0.38	0.013
bta-miR-*19b*	18.66	13.19	19.85	14.34	5.47	5.51	-0.04	1.03	0.367
bta-miR-27b	14.62	13.19	14.00	13.23	1.42	0.77	0.65	0.64	0.034
bta-miR-*33a*	23.01	13.19	23.91	14.34	9.82	9.57	0.25	0.84	0.064
bta-miR-*101*	19.99	14.16	20.55	14.34	5.83	6.21	-0.38	1.30	0.072
bta-miR-*130a*	20.66	13.99	21.04	14.10	6.67	6.94	-0.26	1.20	0.175
bta-miR-*152*	16.51	14.61	16.12	13.97	1.90	2.16	-0.25	1.19	0.546
bta-miR-*196a*	18.22	15.56	18.51	14.65	2.66	3.85	-1.19	2.28	0.018
bta-miR-*204*	21.45	14.16	21.04	14.10	7.29	6.93	0.36	0.78	0.053
bta-miR-*296-3p*	16.85	14.61	16.41	14.65	2.24	1.76	0.48	0.72	0.069
bta-miR-*378*	14.18	14.16	14.97	14.10	0.02	0.87	-0.84	1.80	0.047
